# Harnessing allogeneic NK cells: improving outcomes with tailored donor lymphocyte infusion

**DOI:** 10.1172/JCI160584

**Published:** 2022-06-01

**Authors:** Shannon R. McCurdy

**Affiliations:** Abramson Cancer Center and the Division of Hematology and Oncology, Hospital of the University of Pennsylvania, Philadelphia, Pennsylvania, USA.

## Abstract

Since researchers first began to uncover the mechanisms underlying allogeneic transplantation, the focus has been on T cells. T cells are a major instigator of graft-versus-host disease (GVHD). The clear association between GVHD occurrence and subsequent reduction in relapse supported concentrating on T cells as the masterminds behind graft-versus-tumor (GVT) effects. Recently, an alternative mediator of GVT has taken center stage: natural killer (NK) cells. Part of the appeal of NK cells is their potential to provide antitumor immunity without GVHD. Donor lymphocyte infusion has been the predominant treatment of relapse after allogeneic transplant, but the mix of lymphocytes includes CD8^+^ T cells and, consequently, a substantial risk for GVHD. In this issue of the *JCI*, Shapiro and colleagues developed an adoptive NK cell transfer platform to treat relapse after haploidentical allogeneic transplant. The study demonstrated safety, sought to determine resistance mechanisms, and provided avenues for future research.

## Treating posttransplant myeloid disease relapse

The standard therapy to treat relapse of myeloid diseases after allogeneic transplantation includes chemotherapy to reduce tumor cell volume followed by donor lymphocyte infusion (DLI) or a second allogeneic transplant. Both strategies have similar long-term survival in the realm of 30%, but logistically DLI is easier and does not require as robust a performance status compared with undergoing a second allogeneic transplant. In the early days of haploidentical transplant, physicians avoided DLI due to fear of eliciting severe graft-versus-host disease (GVHD). Subsequent studies have demonstrated that haploidentical DLI is safe at starting doses of 1 × 10^6^ mononuclear cells/kg, likely due to persisting T regulatory cells (Tregs). Although posttransplant cyclophosphamide (PTCy) kills alloreactive T cells, Tregs may be the most important mediator of GVHD prevention in this setting and their presence may prevent severe GVHD after haploidentical DLI (1). Limitations to the use of DLI include risk of GVHD and low response rates, which in HLA-mismatched settings may in part be due to the loss of HLA on malignant cells.

The holy grail of transplantation is the ability to dissect GVHD and graft-versus-tumor (GVT) effects. In the absence of genetic engineering to reduce alloreactivity or focus attacks on a tumor antigen, allogeneic CD8^+^ T cells contained within DLIs may elicit GVHD. In this issue of the *JCI*, Shapiro et al. present an alternative to DLI in the treatment of posttransplant relapse. The strategy involved infusing patients with natural killer (NK) cells followed by IL-2 administration to expand and maintain the adoptively transferred NK cells (2).

The importance of NK cells in prevention of relapse after allogeneic transplant, particularly with HLA-mismatched grafts and PTCy, is being increasingly recognized. NK cell alloreactivity may be more readily harnessed in mismatched HLA settings due to killer Ig-like receptor (KIR) ligands, particularly in T cell–depleted haploidentical transplant where T cells play less of an antitumor role (3, 4). More recent data suggest that regaining a mature NK cell phenotype is associated with less leukemia recurrence in the context of haploidentical transplant with PTCy (5). In both HLA-matched and HLA-haploidentical transplantation with PTCy, we have demonstrated that numerical recovery of NK cells at day 28 was associated with less relapse and nonrelapse mortality (6).

GVHD prophylaxis, such as with PTCy and tacrolimus, may contribute to eradication of mature NK phenotypes and subsequent impaired recovery after transplant, which would limit the power of NK cells to elicit GVT effects. While adoptive NK cell transfer is usually done in the absence of immunosuppression, lack of persistence in vivo and inhibition from Tregs or the tumor itself may block their efficacy. Shapiro and colleagues sought to overcome some of these drawbacks by incubating cells in IL-12, IL-15, and IL-18 to generate cytokine-induced memory-like (CIML) NK cells, which show enhanced in vitro cytotoxicity to leukemic cells (7). A subsequent phase I trial of CIML NK cells from haploidentical donors followed by IL-2 administration for patients with relapsed and refractory acute myeloid leukemia (AML) who had never undergone allogeneic transplantation demonstrated a complete remission (CR) and CR with incomplete hematologic recovery (CRi) rate of 50%. A major drawback to the trial’s initial approach was the lack of NK cell persistence beyond two to four weeks after administration that was attributed to HLA incompatibility between the host and the NK cell donor (8, 9).

## Testing the NK cell transfer platform

To address the lack of NK cell persistence, the authors decided to test their platform in patients who had relapsed after a prior haploidentical transplant, where the engrafted donor immune system is autologous to the adoptively transferred NK cells. This time, Shapiro and colleagues demonstrated the persistence of NK cells for up to several months after the last dose of IL-2. Four out of six patients achieved a response and the NK cells were found to infiltrate sites of disease, including bone marrow and an extramedullary mass in a patient with blastic plasmacytoid dendritic cell neoplasm, with colocalization of CD8^+^ T cells to the tumor sites in several patients. Their subsequent analyses suggested that the expression of NK inhibitory ligands may provide a possible mechanism of relapse after NK cell adoptive therapy.

Shapiro and colleagues chose a haploidentical transplant platform because of the above-mentioned utility of NK cells in mismatched settings and the wide availability of haploidentical donors. However, haploidentical transplant may also represent the ideal scenario to employ NK cell adoptive transfer given the limited efficacy of standard DLI due in part to HLA loss relapse, a phenomenon that occurs in 30% of relapses after haploidentical transplant (10). HLA loss occurs when the mismatched HLA molecules are downregulated or genetically deleted due to copy-neutral loss of heterozygosity (CN-LOH) and can result in immune evasion and subsequent relapse. T cell identification of mismatched HLA molecules may be the main mechanism by which DLI eradicates tumor cells after HLA-mismatched transplant and thus HLA loss may make DLI ineffective. Consequently, several research groups have focused on alternative mechanisms to treat relapse after haploidentical transplant (11, 12). NK cells do not require recognition of HLA for cytotoxicity; therefore, NK cell adoptive transfer may offer a particularly promising treatment strategy for HLA loss relapse, without the GVHD risk that standard DLI possesses.

## An NK cell era

Like Shapiro et al. (2), other researchers have concentrated on the utility of haploidentical NK cells to treat or prevent relapse after haploidentical transplant. Ciurea and colleagues used IL-21 to expand NK cells followed by prophylactic adoptive transfer in high-risk patients, which demonstrated encouraging phase I results (13). Miller and colleagues spearheaded another approach to expand NK cells by administering IL-15 after NK cells, a strategy that resulted in a 32% to 40% remission rate for relapsed and refractory leukemia (outside the context of transplantation) (14). In contrast to Miller’s group, Shapiro and colleagues did not demonstrate substantial GVHD in their study, which they concluded was due to administration of NK cells with IL-2 rather than IL-15 (2). In contrast, the most notable adverse event identified in Shapiro et al. (2) was pancytopenia, which required a CD34^+^ stem cell boost (15) in 2 patients. This prolonged aplasia has also been observed in allogeneic chimeric antigen receptor T cell (CAR-T) recipients.

After sharing their exciting preliminary data on 6 cases of NK cell adoptive transfer with IL-2 expansion to treat relapse after haploidentical transplant, Shapiro et al. (2) conclude that the next research chapter will include adding different immunomodulatory agents to the CIML NK cell platform or to arm NK cells with CAR gene constructs. This latter method may enable NK cell persistence without the need for in vivo cytokine administration, may improve the NK cell longevity, and may allow for off-the-shelf products that do not require HLA matching. Just as in allogeneic transplant, NK cells may represent a new focus in CAR therapy research. Shapiro et al. (2) point to the promise of this next phase of investigation in what may be an NK cell era.

## Version 1 06/01/2022

Electronic publication

## References

[1] Ganguly S (2014). Donor CD4^+^ Foxp3^+^ regulatory T cells are necessary for posttransplantation cyclophosphamide-mediated protection against GVHD in mice. Blood.

[2] Shapiro RM (2022). Expansion, persistence, and efficacy of donor memory-like NK cells infused for posttransplant relapse. J Clin Invest.

[3] Locatelli F (2018). NK cells mediate a crucial graft-versus-leukemia effect in haploidentical-HSCT to cure high-risk acute leukemia. Trends Immunol.

[4] Ruggeri L (2021). Natural killer cell alloreactivity in HLA-haploidentical hematopoietic transplantation: a study on behalf of the CTIWP of the EBMT. Bone Marrow Transplant.

[5] Russo A (2018). NK cell recovery after haploidentical HSCT with posttransplant cyclophosphamide: dynamics and clinical implications. Blood.

[6] McCurdy SR (2022). Signatures of GVHD and relapse after posttransplant cyclophosphamide revealed by immune profiling and machine learning. Blood.

[7] Romee R (2012). Cytokine activation induces human memory-like NK cells. Blood.

[8] Romee R (2016). Cytokine-induced memory-like natural killer cells exhibit enhanced responses against myeloid leukemia. Sci Transl Med.

[9] Keppel MP (2013). Murine NK cell intrinsic cytokine-induced memory-like responses are maintained following homeostatic proliferation. J Immunol.

[10] Crucitti L (2015). Incidence, risk factors and clinical outcome of leukemia relapses with loss of the mismatched HLA after partially incompatible hematopoietic stem cell transplantation. Leukemia.

[11] Imus PH (2017). Major histocompatibility mismatch and donor choice for second allogeneic bone marrow transplantation. Biol Blood Marrow Transplant.

[12] Wu H (2021). Blinatumomab for HLA loss relapse after haploidentical hematopoietic stem cell transplantation. Am J Cancer Res.

[13] Ciurea SO (2017). Phase 1 clinical trial using mbIL21 ex vivo-expanded donor-derived NK cells after haploidentical transplantation. Blood.

[14] Cooley S (2019). First-in-human trial of rhIL-15 and haploidentical natural killer cell therapy for advanced acute myeloid leukemia. Blood Adv.

[15] Shahzad M (2021). Outcomes with CD34-selected stem cell boost for poor graft function after allogeneic hematopoietic stem cell transplantation: a systematic review and meta-analysis. Transplant Cell Ther.

